# Lateral Displacement Calcaneal Osteotomy Combined With Anterolateral Transfer of the Tibialis Posterior Tendon for Treating Flexible Cavovarus Foot in an Adult Patient: A Case Report

**DOI:** 10.7759/cureus.80751

**Published:** 2025-03-18

**Authors:** Dan Moriwaki, Tomoyuki Nakasa, Yasunari Ikuta, Shingo Kawabata, Nobuo Adachi

**Affiliations:** 1 Department of Orthopaedic Surgery, Graduate School of Biomedical and Health Sciences, Hiroshima University, Hiroshima, JPN; 2 Department of Artificial Joints and Biomaterials, Graduate School of Biomedical and Health Sciences, Hiroshima University, Hiroshima, JPN

**Keywords:** calcaneal osteotomy, cavovarus foot, first metatarsal osteotomy, joint preserving technique, tibialis posterior tendon transfer

## Abstract

The cavovarus foot is a spectrum of foot deformity primarily caused by muscular imbalance and characterized by increased pitch, varus of the hindfoot, plantar flexion of the midfoot, and pronation and adduction of the forefoot. Surgery for cavovarus foot commonly involves some combination of procedures, however, no consensus has emerged regarding their choice. We report the case of a 68-year-old male with a flexible cavovarus foot. The patient complained of pain in the subtalar joint and plantar callosities. For this patient, we performed lateral displacement calcaneal osteotomy (LDCO), anterolateral transfer of the tibialis posterior tendon (TPT), and dorsiflexion first metatarsal osteotomy (DFMO). At one year and nine months after surgery, plain radiographs revealed bony union at the osteotomy sites of the calcaneus and first metatarsal. The Japanese Society for Surgery of the Foot hindfoot scale and visual analogue scale improved from 52 points preoperatively to 82 points at the final follow-up and from 3 points to 1 point, respectively. A combination of LDCO, TPT, and DFMO is effective for adult patients with flexible cavovarus foot.

## Introduction

The cavovarus foot is a spectrum of foot deformity characterized by increased pitch, varus of the hindfoot, plantar flexion of the midfoot, and pronation and adduction of the forefoot [1]. It is often caused by muscle imbalances resulting from neurologic, traumatic, residual club foot, or idiopathic etiologies [2]. The combination of weakness of the tibialis anterior muscle relative to the peroneus longus muscle and weakness of the peroneus brevis muscle relative to the tibialis posterior muscle can cause plantarflexion of the first ray and hindfoot varus [3,4]. The overaction of the peroneus longus results in the plantarflexion of the first metatarsal, and the strong tibialis posterior relative to the peroneus brevis exacerbates the hindfoot varus, leading to midfoot supination. This results in the peroneus longus further depressing the first ray to keep the forefoot plantigrade. Cavovarus deformity can cause pain and callosity formation under the metatarsal heads, foot fatigue, difficulty wearing normal shoes, lateral ankle instability, and tripping [5]. Since the underlying disease, deformity site, and severity vary from case to case, individualized treatment is required. Surgical treatment is considered for cases that do not respond to conservative treatment such as physical therapy, cast, insole, and customized shoes. Various surgical procedures for cavovarus deformities have been reported, including soft-tissue release [6,7], tendon transfer [8-10], osteotomy [6,7,11], and arthrodesis [12]. A combination of several procedures is commonly used in surgical treatment for cavovarus foot; however, there is no consensus on the specific procedures, which depend on the surgeon’s preference and experience. Lateral displacement calcaneal osteotomy (LDCO) and anterolateral transfer of the tibialis posterior tendon (TPT) have been reported as surgical options for the cavovarus foot [6,8,13-16]. To the best of our knowledge, there are no reports of LDCO and TPT being performed simultaneously in adult patients with cavovarus foot.

We report a case of an adult flexible cavovarus foot who achieved good clinical outcomes by a combination of LDCO, TPT, and dorsiflexion first metatarsal osteotomy (DFMO).

## Case presentation

A 68-year-old male presented with bilateral cavovarus foot deformity and pain during walking for approximately 20 years. The patient reported a history of some kind of neurological disease since childhood, possibly mild infantile paralysis; however, details of the disease were unclear because he was unfamiliar with it, and we were unable to obtain further information. He had been treated conservatively with medication and an ankle support orthosis at a local clinic; however, the pain in his right foot did not improve. He was referred to our hospital for further treatment.

**Figure 1:**
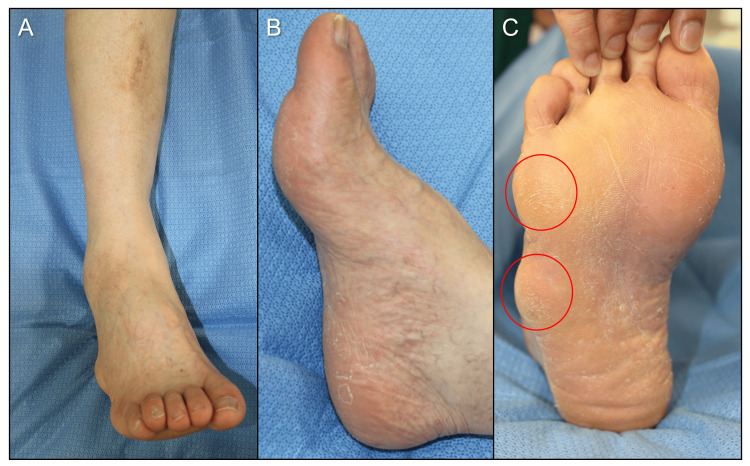
Preoperative appearance of foot. (A) The frontal view of the ankle; (B) the lateral view of the foot; (C) the plantar view (circle indicating callosity).

Physical examination revealed a right flexible cavovarus foot with painful callosities beneath the fifth metatarsal head and the base of the fifth metatarsal (Figures 1A-1C).

**Figure 2:**
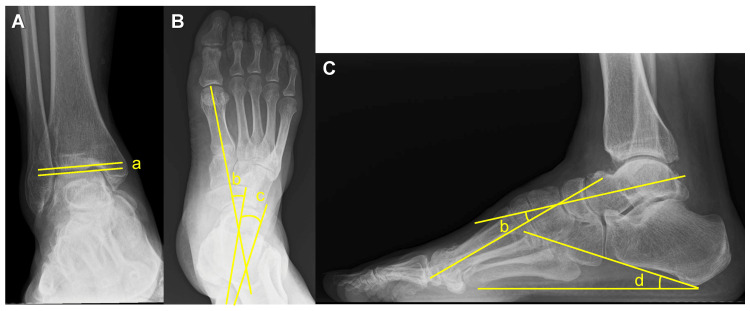
Preoperative plain radiographs on weight-bearing. (A) Anteroposterior view of the ankle; (B) anteroposterior view of the foot; (C) lateral view of the foot. (a) Talar tilt angle; (b) talo-first metatarsal angle; (c) talo-calcaneal angle; (d) calcaneal pitch angle.

The manual muscle test (MMT) around the right ankle was good in dorsiflexion and eversion and normal in plantarflexion and inversion. The contracture of the Achilles tendon was mild. The range of motion (ROM) of the right ankle joint was 10° in dorsiflexion and 40° in plantarflexion. The deformity of the left foot was milder than that of the right foot, and the MMT around the left ankle was almost normal. The patient walked with pain using two canes. Plain radiographs taken during weight-bearing revealed the cavovarus foot deformity, with a talar tilt angle of 0° (normal range: -1° to 1°[17]) on the anteroposterior view of the ankle, a talocalcaneal angle of 8.8° (15° to 27°) and a talo-first metatarsal angle of -16.1° (3° to 11°) on the anteroposterior view of the foot, and a talo-first metatarsal angle of 15.3° (2° to 10°) and a calcaneal pitch angle of 17.4° (13° to 23°) on the lateral view of the foot (Figures 2A-2C).

The Japanese Society for Surgery of the Foot (JSSF) hindfoot scale was 52 points [18], and the Self-Administered Foot Evaluation Questionnaire (SAFE-Q) scores were 68.9 for pain, 43.2 for physical function, 37.5 for social function, 83.3 for shoe-related, and 35.0 for general health [19].

**Figure 3:**
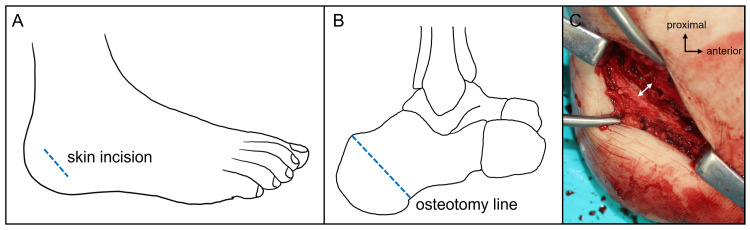
Steps of the medial displacement calcaneal osteotomy. (A) Skin incision on the lateral side of the calcaneus; (B) osteotomy line; (C) the posterior fragment of the calcaneus was displaced laterally (white arrow). Credit: Images (A-B) are created by the authors.

We diagnosed his condition as bilateral paralytic flexible cavovarus foot and decided to proceed with LDCO, TPT, and DFMO to treat the right foot condition. The patient was placed in the supine position, and a thigh tourniquet was applied. First, LDCO was performed through a 4 cm oblique skin incision on the lateral side of the calcaneus (Figure 3A). The calcaneus was exposed, and an oblique osteotomy was made along the incision (Figure 3B). The posterior calcaneal fragment was displaced laterally by about 5 mm (Figure 3C), and the osteotomy site was fixed with a 5.0-mm diameter, 55-mm long cannulated cancellous screw (Ace Medical, El Segundo, CA, USA).

**Figure 4:**
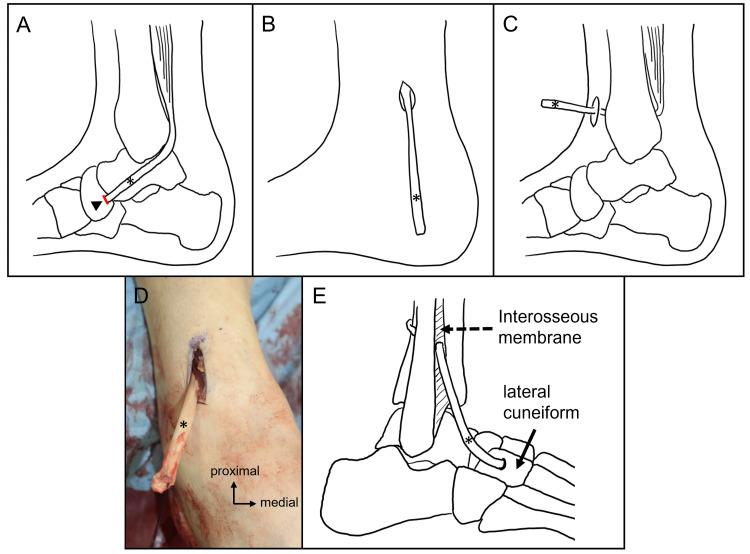
Steps of the transfer of the tibialis posterior tendon. (A) TPT (*) was resected at the insertion (red line) on the navicular bone (arrowhead); (B) TPT was retrogradely pulled out to the incision above the medial malleolus; (C) TPT was passed through a window in the interosseus membrane and pulled out the anterior compartment of the calf; (D) Picture of TPT pulled out the anterior of the calf; (E) TPT was fixed to the osseous tunnel of the lateral cuneiform. TPT: tibialis posterior tendon Credit: Images (A-C & E) are created by the authors.

Next, TPT was performed in four steps. First, a 3-cm longitudinal incision was made over the insertion of the TPT on the navicular bone, and the tendon was resected (Figure 4A). Second, another 3-cm incision was made 8 cm above the medial malleolus. The proximal part of the TPT was exposed, and the entire tendon was retrogradely pulled out to the incision (Figure 4B). Third, a 3-cm longitudinal incision was made along the anterior border of the distal fibula, 5 cm above the ankle joint. The interosseous membrane was incised about 4 cm, and the TPT was passed from medial to lateral, behind the tibia, through the window in the interosseous membrane to the anterior compartment of the calf (Figure 4C). Fourth, a 3-cm incision was made over the lateral cuneiform. The TPT was passed under the retinaculum into the dorsal foot and fixed to the osseous tunnel of the lateral cuneiform using a 4.0-mm diameter interference screw with 10 mm length (BioCompisite Tenodesis Screw, Arthrex, Inc., Naples, USA) (Figure 4D).

Finally, DFMO was performed through a 3-cm longitudinal incision over the first tarsometatarsal (TMT) joint to the midpoint of the first metatarsal. A wedge osteotomy was made 15 mm distal to the first TMT joint. The distal metatarsal fragment was dorsiflexed, and the osteotomy site was fixed with a compression staple (DynaNite, 15 mm x 12 mm, Arthrex, Inc.). A short-leg cast was applied for four weeks. The ROM exercise and strength training of foot and ankle and partial weight-bearing was initiated four weeks postoperatively. Full weight-bearing was permitted seven weeks postoperatively.

**Figure 5:**
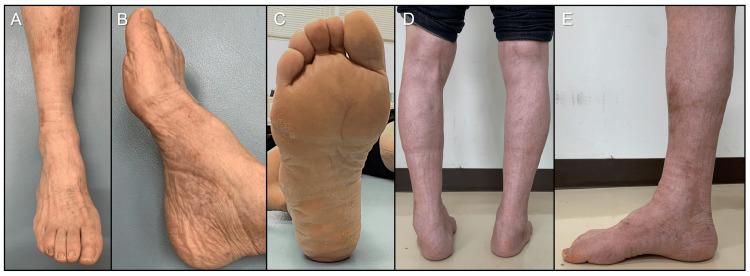
Improvement of the right foot deformity at 20 months after surgery. (A) The frontal view of the ankle; (B) the lateral view of the foot; (C) the plantar view; (D) the posterior view of the bilateral leg in the weight-bearing position; (E) the lateral view of the foot in the weight-bearing position.

During the postoperative course, his symptoms improved and the bony union was confirmed on radiography three months after surgery. Twenty-one months postoperatively, the JSSF hindfoot scale score was 82 points, and the SAFE-Q scores improved to 85.0 for pain, 56.8 for physical function, 79.2 for social function, 100 for shoe-related, and 80.0 for general health; all categories showed improvement. The cavovarus deformity improved, although plantar callosities did not completely disappear (Figures 5A-5C).

**Figure 6:**
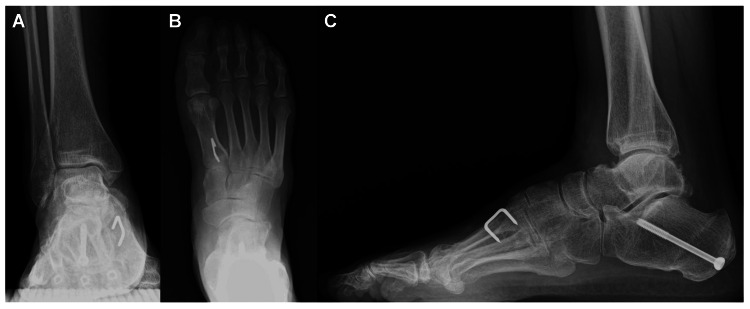
Plain radiographs on weight-bearing at 20 months after surgery. (A) Anteroposterior view of the ankle; (B) anteroposterior view of the foot; (C) lateral view of the foot.

The ROM of the right ankle joint was 20° in dorsiflexion and 40° in plantarflexion, showing an improvement in dorsiflexion after surgery. On plain radiographs taken during weight-bearing, the talar tilt angle remained 0° on the anteroposterior view of the ankle, the talo-calcaneal angle improved to 13.2° and the talo-first metatarsal angle improved to -10.0° on the anteroposterior view of the foot. On the lateral view, the talo-first metatarsal angle improved to 10.7° and the calcaneal pitch angle improved to 15.0°. Bone union was achieved at the osteotomy sites of the calcaneus and first metatarsal (Figures 6A-6C).

## Discussion

Cavovarus foot has long been associated with neurological diseases such as cerebral palsy, Charcot-Marie-Tooth disease, poliomyelitis, spina bifida, or other hereditary sensory and motor neuropathies. According to the patient, he has a history of some neurological disease, possibly infantile paralysis; however, the details remain unclear as we were unable to obtain further information from him. We suspect the deformity to be caused by a long-term muscle imbalance from childhood rather than a progressive neurological disease.

LDCO is a joint-preserving procedure that laterally displaces the attachment of the Achilles tendon and the plantar fascia to correct hindfoot varus. Maskill et al. performed LDCO (100%) in combination with DFMO (86%) and various soft-tissue procedures in 29 feet of 23 patients with subtle flexible cavovarus foot caused by an ankle injury, reporting an improvement in the mean American Orthopaedic Foot and Ankle Society score from 45 to 90 points with 87% (20/23) patient satisfaction [11]. Togei et al. performed LDCO, DFMO, and plantar fasciotomy in all 11 feet of 11 patients, reporting that 91% (10/11) of patients had improvement of painful plantar callosities and significant improvements in postoperative JSSF scale and radiographic outcomes [6]. The amount of displacement is generally limited to no more than 10 mm to reduce the risk of tarsal tunnel syndrome, nerve impingement, and soft tissue complications [20]. Since the amount of displacement is limited, LDCO cannot provide sufficient correction for severe cases. Therefore, LDCO is considered a safe and effective procedure for mild to moderate hindfoot varus.

The tibialis posterior muscle is generally strong and acts as a primary deforming force in the cavovarus foot [15]. TPT serves two goals: first, it reduces the primary deforming forces in the cavovarus foot, thereby decreasing the risk of recurrence; and second, it compensates for weakened tibialis anterior and peroneus brevis muscle function, improving dorsiflexion and valgus forces [7,15]. While fixation of TPT to the intermediate cuneiform is common when dorsiflexion forces are the primary goal, valgus forces can be achieved by fixation to the lateral cuneiform, cuboid, or peroneus brevis tendon [7,9,10]. Chen et al. performed TPT with various osteotomies and soft-tissue releases in 28 feet of 21 children aged 10 to 14 years, 10 with fixation to intermediate cuneiform and 18 with fixation to lateral cuneiform, reporting that 89% (25/28) achieved very good or good clinical results, with significant improvement in radiographic outcomes and no complications [7]. Shen et al. performed TPT to the lateral cuneiform in all 24 feet of 19 pediatric patients with cavovarus foot, reporting all the patients, except one patient who required a secondary procedure, showed improved gait, and all transferred tendons were palpated during active dorsiflexion or withdrawal of the foot without complications [8]. In adult cavovarus foot, the transferred TPT typically does not lead to subsequent planovalgus deformity because the bony structures and ligaments are usually sufficient to maintain the medial arch, preventing collapse [2].

In surgery for the cavovarus foot, joint-preserving surgeries, such as soft-tissue release and osteotomies, are typically performed while the deformity is still flexible [3,6,11], with joint fusion being considered when the deformity becomes rigid [12]. Since the deformity in our case was flexible, joint-preserving surgeries were preferred. We performed LDCO and TPT for hindfoot varus and DFMO for plantarflexion of the first ray. We obtained good clinical outcomes following a combination of LDCO, TPT, and DFMO in an adult patient with a flexible cavovarus foot.

## Conclusions

Cavovarus foot is a complex condition involving multiple foot deformities. It is essential to assess the site and severity of each deformity and select the appropriate surgical procedure for each case. We have described joint-preserving surgeries for an adult patient with a flexible cavovarus foot, and good short-term clinical outcomes were achieved through a combination of LDCO, TPT, and DFMO.
